# Quantitative methodology is critical for assessing DNA methylation and impacts on correlation with patient outcome

**DOI:** 10.1186/1868-7083-6-22

**Published:** 2014-12-09

**Authors:** Annette M Lim, Ida LM Candiloro, Nicholas Wong, Marnie Collins, Hongdo Do, Elena A Takano, Christopher Angel, Richard J Young, June Corry, David Wiesenfeld, Stephen Kleid, Elizabeth Sigston, Bernard Lyons, Danny Rischin, Benjamin Solomon, Alexander Dobrovic

**Affiliations:** Department of Medical Oncology, Peter MacCallum Cancer Centre, Locked Bag 1 A’Beckett Street, Melbourne, Victoria 8006 Australia; The University of Melbourne, Parkville, Victoria 3010 Australia; Research Division, Peter MacCallum Cancer Centre, Locked Bag 1 A’Beckett Street, Melbourne, Victoria 8006 Australia; Department of Microbiology and Immunology, The University of Melbourne, at the Peter Doherty Institute for Infection and Immunity, Melbourne, Victoria 3010 Australia; Translational Genomics and Epigenomics Laboratory, Ludwig Institute for Cancer Research, Olivia Newton-John Cancer & Wellness Centre, Austin Health, 145-163 Studley Road, Heidelberg, Victoria 3084 Australia; Department of Biostatistics and Clinical Trials, Peter MacCallum Cancer Centre, Locked Bag 1 A’Beckett Street, Melbourne, Victoria 8006 Australia; Department of Pathology, Peter MacCallum Cancer Centre, Locked Bag 1 A’Beckett Street, Melbourne, Victoria 8006 Australia; Department of Radiation Oncology, Peter MacCallum Cancer Centre, Locked Bag 1 A’Beckett Street, Melbourne, Victoria 8006 Australia; Department of Surgery, Royal Melbourne Hospital, 300 Grattan St, Parkville, Victoria 3050 Australia; Department of Surgical Oncology, Peter MacCallum Cancer Centre, Locked Bag 1 A’Beckett Street, Melbourne, Victoria 8006 Australia; Department of Surgery, Monash Medical Centre, 246 Clayton Road, Clayton, Victoria 3168 Australia; Department of Surgery, St Vincent’s Hospital, PO Box 2900, Fitzroy, Victoria 3065 Australia; Department of Pathology, The University of Melbourne, Parkville, Victoria 3010 Australia; School of Cancer Medicine, La Trobe University, Bundoora, Victoria 3084 Australia

**Keywords:** Quantitative, Methylation, Head and neck cancer, *RUNX3*, Tongue

## Abstract

**Background:**

DNA hypermethylation is reported as a frequent event and prognostic marker in head and neck squamous cell carcinomas (HNSCC). Methylation has been commonly assessed with non-quantitative methodologies, such as methylation-specific PCR (MSP). We investigated previously reported hypermethylated genes with quantitative methodology in oral tongue squamous cell carcinomas (OTSCC).

**Results:**

The methylation status of 12 genes in 115 OTSCC samples was assessed by one or more of three quantitative analyses: methylation sensitive high resolution melting (MS-HRM), sensitive-melting analysis after real time-methylation specific PCR (SMART-MSP), and bisulfite pyrosequencing.

In contrast to much of the literature, either no or infrequent locus-specific methylation was identified by MS-HRM for *DAPK1*, *RASSF1A*, *MGMT*, *MLH1*, *APC, CDH1*, *CDH13, BRCA1*, *ERCC1*, and *ATM*. The most frequently methylated loci were *RUNX3* (18/108 methylated) and *ABO* (22/107 methylated). Interrogation of the Cancer Genome Atlas (TCGA) HNSCC cohort confirmed the frequency of significant methylation for the loci investigated.

Heterogeneous methylation of *RUNX3* (18/108) and *ABO* (22/107) detected by MS-HRM, conferred significantly worse survival (*P* = 0.01, and *P* = 0.03). However, following quantification of methylation levels using pyrosequencing, only four tumors had significant quantities (>15%) of *RUNX3* methylation which correlated with a worse patient outcome (*P* <0.001), while the prognostic significance of *ABO* hypermethylation was lost. *RUNX3* methylation was not prognostic for the TCGA cohort (*P* = 0.76).

**Conclusions:**

We demonstrated the critical need for quantification of methylation levels and its impact on correlative analyses. In OTSCC, we found little evidence of significant or frequent hypermethylation of many loci reported to be commonly methylated. It is likely that previous reports have overestimated the frequency of significant methylation events as a consequence of the use of non-quantitative methodology.

**Electronic supplementary material:**

The online version of this article (doi:10.1186/1868-7083-6-22) contains supplementary material, which is available to authorized users.

## Background

DNA methylation, which is characterized by 5-methylcytosines in CpG dinucleotides, can mediate an epigenetic mechanism of altering gene expression. This epigenetic change provides both an opportunity for therapeutic manipulation, as well as serving as a candidate predictive or prognostic marker [1]. For example, DNA methylation of *MGMT* is considered as both a prognostic marker for patients with glioblastomas as well as a predictive marker for benefit of the use of the DNA alkylating agent, temozolomide, in the management of this disease [2]. Additionally, the use of demethylating agents has transformed the standard management of high-risk myelodysplastic disorders [3].

As epimutations are more frequent than genetic mutations in the cancer genome [4], DNA methylation forms an ideal putative biomarker. Indeed, DNA hypermethylation is a commonly reported phenomenon for multiple genes in head and neck squamous cell carcinomas (HNSCC). For example, *CDKN2A* disruption is recognized as an early event in carcinogenesis of HNSCC that can arise due to promoter methylation [5], and additionally may be predictive of disease recurrence [6, 7]. Many other genes have been reported to be frequently methylated across head and neck subsites, including *DAPK1, RASSF1A* and *MGMT* [8–10]. However, considerable variation in the frequency of reported methylation events for most loci is described, and correlation with clinicopathological features within heterogeneous cohorts is unclear.

In addition, the common use of non-quantitative methodology hinders the interpretation of the relevance of reported hypermethylated loci. Non-quantitative methodology is prone to the inclusion of false positive results [11–15]. Furthermore, the failure to quantify methylation incorrectly assumes homogeneity of levels and the significance of all detected methylation [14, 15]. One of the most frequently used, non-quantitative techniques is methylation-specific polymerase chain reaction (MSP), which relies only on gel electrophoresis resolution of PCR products to determine the presence of methylation [16].

Thus, in order to identify putative prognostic markers for oral tongue squamous cell carcinomas (OTSCC), we assessed a panel of literature-identified genes reported to be hypermethylated in HNSCC with only quantitative methodology, and we sought to determine any correlation with patient outcome. The loci interrogated included the promoter region of the DNA repair genes: *MGMT*, *MLH1*, *ATM*, *BRCA1* and *ERCC1* [6, 9, 10, 17–20]; genes mediating control of cellular proliferation: *RASSF1A, APC* and *RUNX3* [8, 9, 21–23]; the pro-apoptotic tumor suppressor gene: *DAPK1* [6, 9, 10, 24] *;* and genes involved in invasion and metastases: *CDH1*, *CDH13* and *ABO* [9, 24–27]. Table 1 provides a summary of the HNSCC literature in terms of the frequency of methylation events for each gene, the methodology used for the detection of methylation, and the univariate correlative analysis with patient survival.

**Table 1:** **Literature identified hypermethylated loci in head and neck squamous cell carcinomas, according to frequency, methodology and univariate correlation with survival** ^a^

Gene	% Methylation (***n***)	Publication	Methodology ^b^	Correlation with outcome ^c^
***ABO***	**33** (30)	[28]	MSP, MCA	-
***APC***	**9-71%**			
	13 (47)	[29]	MSP	-
	17 (79)	[30]	MS-MLPA	N
	71 (84)^d^	[31]	MSP	-
	15 (34)	[22]	MSP, MCA	-
	18 (77)	[27]	Nested MSP	N
	9 (126)^e^	[32]	MS-MLPA	-
***ATM***	**0-88%**			
	42 (24)^f^	[33]	MSP	-
	88 (84)^d^	[31]	MSP	-
	0 (37)	[34]	Pyrosequencing	-
	25 (100)	[18]	MSP	W
	1 (126 )^e^	[32]	MS-MLPA	-
***BRCA1***	**0-95%**			
	95 (58)^d^	[20]	Pyrosequencing	-
	0 (89)	[35]	MSP	-
	0 (126)^e^	[32]	MS-MLPA	-
***CDH1***	**0-88%**			
	0 (32)	[36]	MSP	-
	18 (48)	[37]	qMSP	N
	88 (43)	[38]	MSP	-
	33 (340)^f^	[39]	MSP	I
	13 (38)	[40]	MSP	I
	43 (54)	[41]	MSP	-
	62 (76)	[42]	MSP	-
	64 (86)	[25]	RE-MSP	W^g^
	78 (23)	[43]	MSP	-
	43 (190)	[9]	MSP	N
	42 (47)	[29]	MSP	-
	43 (77)	[27]	Nested MSP	W
	38 (37)	[34]	Pyrosequencing	-
	42 (79)	[44]	Pyrosequencing	-
	36 (80)	[45]	MSP	-
	66 (33)^h^	[46]	qMSP	N
	35 (99)	[47]	RE-MSP	-
	35 (55)^f^	[48]	MSP	-
***CDH13***	**10-34%**			
	34 (79)	[30]	MS-MLPA	N
	10 (126)^e^	[32]	MS-MLPA	-
***CDKN2A*** **(P16)**	**0-95%**			
	38 (56)	[49]	MSP	-
	49 (73)	[50]	MSP	N
	36 (47)	[51]	MSP	N
	49 (51)	[52]	MSP	-
	12 (61)	[53]	MSP	N
	48 (92)	[54]	qMSP	-
	22 (48)	[37]	qMSP	N
	78 (40)^h^	[55]	MSP	N
	13 (79)	[30]	MS-MLPA	N
	63 (43)	[38]	MSP	-
	5 (42)	[40]	MSP	N
	60 (54)	[41]	MSP	-
	87 (38)	[7]	MSP	-
	17 (24)^f^	[56]	MSP	-
	27 (56 )^h^	[57]	RE-MSP	-
	79 (75)	[58]	MSP	I
	20 (20)	[59]	MSP	-
	44 (126)	[9]	MSP	N
	59 (47)	[29]	MSP	-
	58 (77)	[27]	Nested MSP	N
	95 (41)	[60]	MSP	-
	26 (37)	[34]	Pyrosequencing	-
	28 (79)	[44]	Pyrosequencing	-
	24 (45)^f^	[61]	MSP	-
	20 (20)	[62]	RE-MSP	-
	33 (80)	[45]	MSP	-
	29 (96)	[63]	MSP^i^	N
	47 (30)	[10]	MSP	-
	29 (116)	[64]	MSP	W
	27 (95)	[65]	MSP	-
	20 (121)	[66]	MSP	-
	29 (52)^h^	[46]	qMSP	W
	23 (99)	[47]	RE-MSP	N
	8 (51)^f^	[48]	MSP	-
	23 (30)	[67]	RE-MSP	-
	0 (126)^e^	[32]	MS-MLPA	-
***DAPK1***	**7-81%**			
	14 (79)	[30]	MS-MLPA	N
	81 (43)	[38]	MSP	-
	11 (44)	[40]	MSP	N
	60 (54)	[41]	MSP	-
	76 (41)	[68]	MSP	-
	42 (290)	[9]	MSP	N
	37 (77)	[27]	Nested MSP	N
	30 (47)	[29]	MSP	-
	19 (32)	[36]	MSP	-
	45 (20)	[59]	MSP	-
	39 (18)	[60]	MSP	-
	24 (80)	[45]	MSP	-
	7 (96)	[63]	MSP^i^	N
	33 (30)	[10]	MSP	-
	18 (95)	[65]	MSP	-
	42 (33)^h^	[46]	qMSP	N
	20 (49)^f^	[48]	MSP	-
	12 (126)^e^	[32]	MS-MLPA	-
***ERCC1***	**51** (84)	[19]	MSP	N
***MGMT***	**18-74%**			
	30 (47)	[51]	MSP	N
	53 (51)	[52]	MSP	-
	10 (20)	[53]	Pyrosequencing	W^j^
	30 (88)	[53]	MSP	W^j^
	36 (44)^h^	[69]	MSP	-
	31 (32)	[36]	MSP	-
	43 (40)	[40]	MSP	N
	53 (54)	[41]	MSP	-
	74 (76)	[42]	MSP	-
	50 (20)	[59]	MSP	-
	27 (212)	[9]	MSP	N
	54 (41)	[68]	MSP	-
	31 (37)	[34]	Pyrosequencing	-
	28 (21)	[70]	MSP	-
	23 (30)	[10]	MSP	-
	33 (95)	[65]	MSP	-
	21 (33)^h^	[46]	qMSP	N
	38 (47)	[29]	MSP	-
	34 (77)	[27]	Nested MSP	N
	41 (99)	[47]	RE-MSP	-
	25 (52)^f^	[48]	MSP	-
	18 (94)	[71]	MSP	W
***MLH1***	**0-88%**			
	26 (47)	[29]	MSP	-
	18 (28)	[72]	MSP	-
	0 (20)	[73]	RE-MSP	-
	23 (62)	[49]	MSP	-
	23 (47)	[51]	MSP	N
	47 (116)^e^	[74]	RE-MSP	-
	37 (123)	[75]	RE-MSP	-
	88 (8)	[76]	MSP	-
	69 (54)	[41]	MSP	-
	0 (37)	[34]	Pyrosequencing	-
	29 (49)	[17]	MSP	-
	76 (50)	[77]	MSP	-
	0 (96)	[63]	MSP^i^	N
	8 (99)	[47]	RE-MSP	-
	14 (43)^f^	[48]	MSP	-
	0 (126)^e^	[32]	MS-MLPA	-
***RASSF1A***	**0-44%**			
	17 (24)	[78]	RE-MSP	-
	12 (66)	[53]	MSP	N
	2 (41)	[40]	MSP	N
	18 (54)	[41]	MSP	-
	0 (32)	[36]	MSP	-
	32 (41)	[68]	MSP	-
	38 (47)	[27]	MSP	-
	44 (18)	[60]	MSP	-
	22 (482)^h^	[8]	dHPLC	N
	8 (80)	[45]	MSP	-
	33 (33)^h^	[46]	qMSP	N
	6 (50)^f^	[48]	MSP	-
	11 (126)^e^	[32]	MS-MLPA	-
***RUNX3***	**18-70%**			
	70 (30)	[79]	MSP	-
	26 (47)	[29]	MSP	-
	25 (76)	[21]	MSP	N
	18 (45)^f^	[61]	MSP	-

^a^Methylation frequency is only reported for intra-tumoral methylation. A summary of the range of reported methylation is presented in bold font when available.

^b^Methodology: dHPLC, denaturing high-performance liquid chromatography; MCA, melting curve analysis; MSP, methylation-specific PCR; MS-MLPA, methylation specific multiplex ligation-dependent probe amplification; RE-MSP, restriction enzyme MSP. A prefixed ‘q’ indicates the use of a quantitative version of the methodology.

^c^Correlation with outcome: W, Worse; I, Improved; N, None; - Not performed.

^d^these studies identified similar frequencies or quantities of methylation as control samples.

^e^these studies included samples with mixed histotypes, not just squamous cell carcinomas.

^f^these studies tested for the presence of the Human Papillomavirus.

^g^this study determined correlation with outcome based on immunohistochemistry.

^h^these studies include patients with betel nut or areca nut exposure.

^i^these studies quantified the band intensity on gel electrophoresis.

^j^this study examined a subset of samples that had demonstrated methylation with MSP, with quantitative methodology.

The variety of loci has been selected for two reasons; firstly, to investigate the large variation in the reported frequency of methylation events, and secondly, to investigate loci relevant to carcinogenesis. Specifically, *APC, ATM, CDH1, CDKN2A, DAPK1, MLH1, MGMT*, and *RASSF1A* were chosen due to the high frequency of methylation events reported in the literature and their plausible roles in HNSCC carcinogenesis. In addition, other loci of relevance to cancer were selected. As concurrent cisplatin-based chemoradiotherapy is a standard of care for advanced HNSCC, methylation of *ERCC1* was investigated due to reports of the prognostic significance of expression of this nucleotide excision repair protein in other head and neck subsites (laryngeal and nasopharyngeal) [80, 81] and its association with resistance to cisplatin therapy [81–83]. *ERCC1* has also been reported by us as being methylated in low frequency in lung cancer [84]. *ABO* was selected due to reports specifically in oral carcinomas that detected methylation of the gene, loss of expression of these epithelial antigens [28], and frequent alteration of chromosome 9q34 where the *ABO* gene resides [85, 86]. Methylation of *ATM* was investigated due to a number of reasons: patients who suffer ataxia telangiectasia defined by *ATM* gene dysfunction are known to be predisposed to the development of oral carcinomas [87]; there are reports demonstrating high frequencies of *ATM* methylation in HNSCC [18, 31, 33]; *ATM* plays a crucial role in double-stranded DNA repair, and thus, gene dysfunction contributes to exquisite sensitivity to radiation treatment, which is a key therapeutic modality for the management of HNSCC [88, 89]; and due to our previous investigation of the impact of other mechanisms of *ATM* gene loss in HNSCC [90]. *BRCA1* was investigated due to its role in homologous DNA repair, and as part of the Fanconi Anemia/*BRCA* pathway [35, 91, 92], with the known genetic predisposition of patients with Fanconi Anemia to develop malignancies including oral tongue and oral cavity squamous cell carcinomas [93]. *CDH13* was of interest as part of the cadherin family, with reports suggesting that methylation of this gene was relevant in HNSCC [24, 30, 32, 94]. Additional reports suggest that methylation of *CDH13* has a prognostic role in other smoking-related carcinomas [95].

Therefore, in a uniform head and neck subsite, the oral tongue, this study used quantitative methodologies to investigate a panel of genes previously reported to be hypermethylated in HNSCC.

## Results

### Clinical details

Details of the full cohort of 131 patients have previously been described [96]. The clinical and tumor characteristics of the 115 patients with bisulfite modified DNA available for methylation analyses are presented in Table 2. Median time of follow-up for the whole cohort was 5.1 (range 0.5 to 9.8) years.

**Table 2:** **Summary of patient and tumor characteristics for the cohort analyzed**

	***N***	%
**Gender**		
Female	41	36%
Male	74	64%
**Age at diagnosis (in years)**		
Mean	57.4	
Standard deviation	15.5	
Median	56	
Range	21 – 93	
<40 years	14	12%
40 to 49 years	24	21%
50 to 59 years	21	18%
60 to 69 years	26	23%
70 to 79 years	24	21%
80+ years	6	5%
**Stage**		
1	33	29%
2	34	30%
3	13	11%
4	35	30%
**T-category**		
1	39	34%
2	45	39%
3	12	10%
4	19	17%
**N-category**		
0	74	64%
1	11	10%
2	30	26%
**Smoking history**		
No/Never	33	30%
Yes	78	70%
Unknown	4	
**Alcohol consumption >20 g/day**		
No/Never/Social	70	63%
Past or current	41	37%
Unknown	4	
**ECOG performance status**		
0	69	61%
1	34	30%
2	7	6%
3	3	3%
Unknown	2	

ECOG, Eastern Cooperative Oncology Group; N-category, Node category; T-category, Tumor category.

### Methylation Sensitive High Resolution Melting (MS-HRM) analyses

A summary of the methylation sensitive high resolution melting (MS-HRM) results for the panel of interrogated genes is presented in Table 3, including results from our previous study on *CDKN2A* [96].

**Table 3:** **Summary of methylation events for each locus assessed by methylation sensitive high resolution melting (MS-HRM), and assessed in the Cancer Genome Atlas (TCGA) cohort** ^a^

Gene	Total number of samples	Level of methylation						TCGA HNSCC(n = 305)	TCGA OTSCC(n = 86)
		0%	<10%	10 to 25%	25 to 50%	>50%	Heterogeneous	β-value >0.2	β-value >0.2
***ABO***	107	78 (73%)	7 (7%)	3 (3%)	0	0	19 (17%)	139 (46%)	32 (37%)
cg13506600, cg07241568									
***APC***	105	102 (97%)	0	0	3 (3%)	0	0	50 (16%)	12 (14%)
cg14479889, cg03667968, cg16970232									
***ATM***	48	48 (100%)	0	0	0	0	0	0	0
cg18457775, cg06750635									
***BRCA1***	45	44 (98%)	1 (2%)	0	0	0	0	0	0
cg20187250, cg15419295, cg16963062, cg16630982, cg21253966, cg04110421, cg04658354									
***CDH1***	114	91 (80%)	10 (9%)	0	0	0	13 (11%)	1 (0%)	1 (0%)
cg16739895, cg23989635, cg11255163									
***CDH13***	111	107 (96%)	1 (1%)	0	0	0	3 (3%)	41 (13%)	9 (10%)
cg08747377									
***CDKN2A*** [96]	113	78 (69%)	15 (13%)	8 (7%)	6 (5%)	6 (5%)	0	55 (18%)	21 (24%)
cg04026675, cg13601799									
***DAPK1***	91	87 (96%)	4 (4%)	0	0	0	0	46 (15%)	8 (9%)
cg08797471, cg13932603									
***ERCC1***	47	47 (100%)	0	0	0	0	0	11 (4%)	3 (3%)
cg20467502, cg23902435									
***MGMT***	50	37 (74%)	13 (26%)	0	0	0	0	23 (8%)	5 (6%)
cg02941816, cg05068430									
***MLH1***	49	49 (100%)	0	0	0	0	0	1 (0%)	0
cg23658326									
***RASSF1A***	49	48 (98%)	1 (2%)	0	0	0	0	11 (4%)	0
cg04743654, cg27569446									
***RUNX3***	108	90 (83%)	0	0	0	0	18 (17%)	37 (12%)	16 (19%)
cg19590532, cg06377278									

The frequency of methylation events assessed by MS-HRM is presented with the frequency of methylation events (β-value >0.2) for the TCGA HNSCC cohort and TCGA OTSCC subgroup. The Cg number of the probes from the Infinium HumanMethylation450 beadchip array that were examined in reference to the MS-HRM amplicons is annotated for each gene as ‘cg locus number’.

TCGA HNSCC, The Cancer Genome Atlas head and neck squamous cell carcinoma cohort; TCGA OTSCC, The Cancer Genome Atlas oral tongue squamous cell carcinoma cohort.

In comparison to much of the previously published literature, no methylation or infrequent methylation was detected for the majority of loci examined. No significant methylation (0%) was detected in any of the samples for the following genes: *DAPK1* (91/91), *RASSF1A* (49/49), *MGMT* (50/50), *MLH1* (49/49), *BRCA1* (45/45), *ERCC1* (47/47) and *ATM* (48/48). For the following loci, the majority of samples had no methylation, but definite methylation was present in a subset of samples: *RUNX3* (18/108 methylated), *ABO* (22/107 methylated), *APC* (3/105 methylated), *CDH1* (13/114 methylated) and *CDH13* (4/112 methylated). Both homogeneous and heterogeneous methylation, as ascertained by melting profiles, was observed for these loci.

Heterogeneous melting patterns were detected for *ABO* (19/107), *CDH1* (13/114), *CDH13* (3/112) and *RUNX3* (18/108). Homogeneous melting patterns were observed for *APC* in three samples (3/105), all of which had a methylation level of greater than 25%. Homogeneous methylation of *ABO* was also observed for three samples, which all demonstrated methylation levels of 10 to 25%. Including the samples with heterogeneous methylation, the frequency of methylation events for *ABO* and *RUNX3* were similar to the literature although numerically less [21, 26].

### Sensitive-melting analysis after real time-methylation specific PCR (SMART-MSP)

On the basis of reports suggesting frequencies of methylation greater than 20% for both *DAPK1* and *RASSF1A* [8, 9, 68] *,* sensitive-melting analysis after real time-methylation specific PCR (SMART-MSP) analyses were used to confirm a subset of the MS-HRM findings [97]. Utilizing this quantitative modification of MSP permitted the concurrent investigation of possible reasons for discordance between MS-HRM results and the MSP-based literature. SMART-MSP incorporates the use of a DNA intercalating dye, which permits both real-time quantification and melting curve analysis (MCA), which can minimize the number of false positives [97].

**Figure 1:**
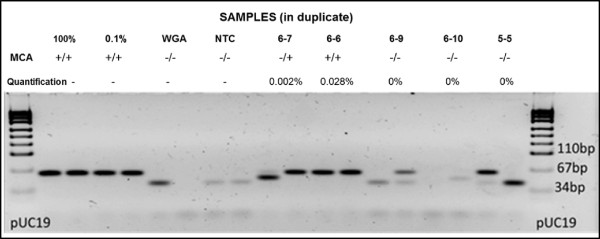
**Sensitive-melting analysis after real time-methylation specific PCR (SMART-MSP) assessment of** ***DAPK1*** **.** Gel electrophoresis of representative SMART-MSP products in duplicate, assessing methylation of the *DAPK1* promoter 1 region. High resolution melting curve analysis (MCA) is represented by + (indicating methylation) and - (indicating no methylation). Quantification of methylation is reported in %. (100% - fully methylated control, 0.1% - 0.1% dilution control, WGA - unmethylated control, NTC – non-template control). The band sizes observed for the 100% methylated control and 0.1% control were appropriately of identical size, due to the inability of this methodology to differentiate quantities of methylation. Sample 6-7 and sample 6-6 demonstrated the presence of methylation on MCA, with gel electrophoresis of PCR products producing band sizes similar to the methylated controls. However, when quantified, the levels of methylation measured below 0.1%. For sample 6-9 and sample 5-5, bands similar to the methylated controls were observed, despite MCA and quantification indicating no detectable levels of methylation. These bands represent false positive results.

The promoter status of *DAPK1* for 50 samples was investigated using SMART-MSP. On quantification, the median level of methylation present was 0.0015% (range 0 to 1.3%), with only one sample demonstrating a level of methylation above 1%. However, despite the low levels of methylation quantified, on MCA analyses 41/50 samples demonstrated a pattern consistent with the presence of methylation, emphasizing the sensitivity of MSP. Gel electrophoresis of SMART-MSP products was also performed (Figure 1), and positive bands were detected for samples with calculated quantification of methylation below 0.2%. False positive, similar sized bands were also observed for some samples that failed to amplify during PCR and also for samples with a MCA pattern consistent with unmethylated DNA. SMART-MSP analysis for *RASSF1A* was performed for ten samples, and no (0%) methylation was detected in these.

Thus, we confirmed with a second quantitative methodology that no significant level of DNA promoter methylation for *DAPK1* and *RASSF1A* was found in the cohort examined. Gel electrophoresis revealed similar sized bands for samples with extremely low levels of methylation or no methylation, illustrating a likely cause in the literature for overestimation of significant methylation events.

### Clinical correlation between methylation sensitive high resolution melting (MS-HRM) analyses and patient outcome

Analyses correlating the presence of promoter methylation with patient outcome was possible only for the following genes for which methylation was detected; *RUNX3, CDH13, APC, ABO* and *CDH1.* Patients with promoter methylation detected for *RUNX3* (18/108, all heterogeneously methylated) had a significantly worse overall survival (OS) compared with patients without methylation, with a five-year OS rate of 32% versus 57% (HR = 2.22, 95% CI: 1.15-4.29, *P* value = 0.01). This effect was maintained for progression-free survival (PFS) (33% versus 51% five-year PFS; HR = 2.07, 95% CI: 1.03-4.18, *P* value = 0.04).

While a much smaller number of events was observed for samples demonstrating *ABO* promoter hypermethylation, for the 3/87 patient samples that demonstrated homogeneous methylation, this significantly correlated with a worse five-year OS rate of 0% versus 57% (HR = 6.51, 95% CI: 1.94-21.8, *P* value = 0.014). Similar results were found for PFS. The effect was maintained when samples with heterogeneous methylation were included in the analysis (3/107, 10 to 25% homogeneously methylated and 19/107 heterogeneously methylated), with a worse five-year OS rate of 32% versus 57% for methylated samples (HR = 1.98, 95% CI: 1.06-3.70, *P* value = 0.03). Significant correlation with PFS was not observed.

### Bisulfite pyrosequencing analyses and correlation with patient outcome

Given the statistically significant correlation with outcome for patient samples with methylation of *RUNX3* and *ABO*, which included unquantified heterogeneously methylated samples, we sought to investigate this further with bisulfite pyrosequencing.

**Figure 2:**
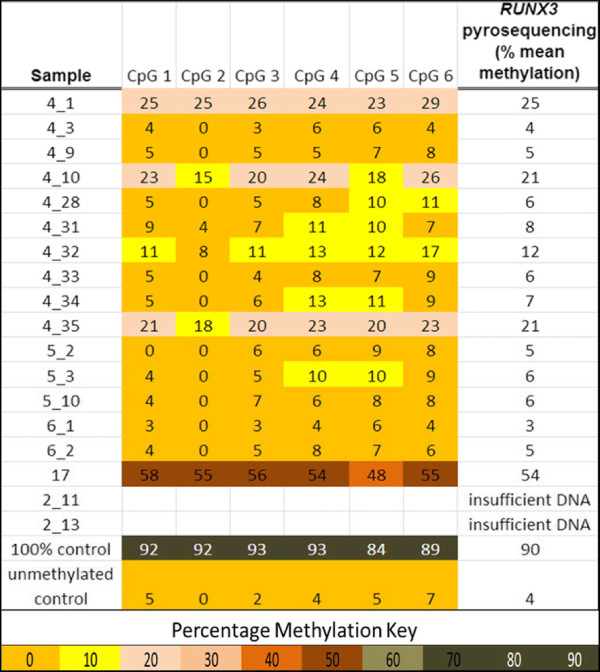
**Pyrosequencing results for all samples demonstrating heterogeneous methylation of** ***RUNX3*** **when assessed by methylation sensitive high resolution melting (MS-HRM).** Each analyzed sample is annotated for the quantity of methylation detected at each CpG site interrogated, and the mean methylation value for all CpG sites analyzed is also provided. This figure demonstrates that the vast majority of samples with heterogeneous methylation for this locus assessed by MS-HRM, had low levels of methylation when quantified by pyrosequencing.

For the *RUNX3* pyrosequencing analysis, 83 samples had sufficient bisulfite modified DNA available for assessment. These samples included the 16/18 samples with heterogeneous methylation identified by MS-HRM. Five samples that failed to produce an adequate signal on pyrosequencing were not included in the analysis. On pyrosequencing, only four samples (4/78) demonstrated mean methylation quantities greater than 15%. The rest of the cohort (74/78), including samples that previously had heterogeneous methylation identified on MS-HRM, had mean quantities of methylation below 12% and thus were scored as negative according to our predefined criteria (Figure 2). A significantly worse outcome was found for the four patients with *RUNX3* promoter hypermethylation greater than 15%, with a five-year OS rate of 0% versus 56% (HR = 10.2, 95% CI: 3.23-32.4, *P* value = 0.001). Given the small number of observed events, firm conclusions could not be made despite the significance of the *P* value, and further analyses were not pursued.

For the *ABO* pyrosequencing analysis, 85 samples had sufficient bisulfite modified DNA available. Of these, 20/85 samples demonstrated levels of mean methylation greater than 15%. For the statistical analysis, two samples that had demonstrated quantities of methylation between 10 to 25% on MS-HRM but had insufficient DNA for pyrosequencing were also included. For the 22 patients with significant methylation of *ABO* identified, no statistically significant clinical correlation with survival was found (HR = 1.43, 95% CI: 0.73-2.80, *P* value = 0.29).

Thus, we demonstrate the impact of quantification of methylation levels. For the investigated loci, though heterogeneous methylation was identified on MS-HRM, the vast majority of these indicated low level methylation events. Of particular note, the initial MS-HRM analysis indicated a frequency of (heterogeneous) *RUNX3* methylation of 17% of samples consistent with the literature. However, when quantified by pyrosequencing, the actual frequency of significant methylation events for *RUNX3* was considerably less (5%). The presence of *RUNX3* promoter methylation was found to correlate with a worse patient outcome, although due to the small numbers robust conclusions cannot be made. In contrast, when *ABO* promoter methylation was quantified with pyrosequencing, correlation with patient outcome was lost with a minor alteration in the frequency of observed events (22/107, 21% for MS-HRM, and 22/85, 26% for pyrosequencing). These observations highlight the impact of methodology and sample size on correlative analyses.

### External validation of results through interrogation of the Cancer Genome Atlas (TCGA) database

To externally validate our findings, which suggested that the frequency of significant methylation events of selected loci was less common than the literature suggested, the Cancer Genome Atlas (TCGA) head and neck cohort were examined. At the time of download (24 June 2013; clinical data download date 22 July 2013), there were 373 TCGA head and neck tumor samples with publically available Infinium HumanMethylation450 beadchip array (HM450K) data available. A total of 26 patients were excluded due to the presence of metastatic disease or an unknown “M-category” (metastasis-category), a history of previous malignancy, or primary site documented as the ‘lip’. Of the remaining 346 patients, 305 patients had both clinical details and methylation data available. Of these 305 patients, 86 patients had OTSCC.

The summary of significant methylation events (β value >0.2) for the examined loci within the TCGA HNSCC cohort is presented in Table 3. For the HM450K probes that overlapped the MS-HRM amplicons, a similar frequency of methylation was observed (see Additional file 1: Figure S1). Methylation levels from probes that flanked the MS-HRM amplicons also indicated a similar frequency of methylation events (see Additional file 1: Figure S2), although comparison across different regions is difficult.

Interestingly, *DAPK1* methylation for the probes flanking the MS-HRM amplicon demonstrated a frequency of methylation events of 15% in the heterogeneous HNSCC TCGA cohort and 9% in the TCGA OTSCC subset. Samples with *DAPK1* methylation in the whole HNSCC cohort were distributed between multiple head and neck subsites. Methylation of *RASSF1A* was identified in 4% of samples in the HNSCC TCGA cohort, but none of these were OTSCC samples.

To assess whether *RUNX3* promoter methylation had prognostic value in the TCGA cohort, methylation reported from the probes flanking the MS-HRM amplicon was correlated with patient outcome for the whole TCGA HNSCC cohort, and for the subset of patients with OTSCC. There were no statistically significant relationships between methylation and survival identified, when methylation from individual probes or methylation averaged across the two probes were used (HR = 1.08, 95% CI: 0.65-1.82, *P* = 0.76 for averaged methylation for the whole TCGA cohort, and HR = 1.27, 95% CI: 0.56-2.88, *P* = 0.57 for averaged methylation for only the OTSCC TCGA subset).

Thus, in an independent, external heterogeneous HNSCC cohort assessed by an orthogonal quantitative methodology, we confirmed the infrequent methylation events for the loci examined. *RUNX3* methylation was not a prognostic marker in the TCGA cohort.

## Discussion

Due to the known limitations of non-quantitative methodology, we evaluated biomarkers frequently reported to be hypermethylated in HNSCC with three quantitative methodologies, in order to identify meaningful correlations with patient outcome. In contrast to much of the published literature, the majority of loci interrogated in our cohort displayed infrequent or no significant methylation. Independent external validation of these findings was provided by the TCGA cohort of HNSCC samples that were assessed by an orthogonal quantitative methodology. Our findings suggest that previous reports may have overestimated the frequency of significant methylation events with the use of non-quantitative but highly sensitive methodology. Furthermore, this study highlights that the use of quantitative methodology to detect significant methylation events impacts upon the identification of meaningful clinicopathological correlations.

In HNSCC, DNA methylation of a wide number of genes has been reported, with considerable variation in the frequency of detected events. For example, promoter methylation of *DAPK1* and *RASSF1A* have been reported to occur with frequencies between 7 and 81%, and 0 and 44%, respectively, most commonly assessed by non-quantitative MSP (Table 1) [9, 27, 36, 38, 45, 60, 63, 68]. Whether variability has arisen due to methodological or anatomical subsite differences is difficult to assess due to the use of heterogeneous cohorts. For example, in a cohort of 41 laryngeal squamous cell carcinomas, 76% (31/41) of the samples demonstrated methylation of *DAPK1* and 32% (13/41) of the samples demonstrated methylation of *RASSF1A* [68]. In comparison, in 47 oral cavity tumors, a frequency of 30% for *DAPK1* and 38% for *RASSF1A* were reported [29]. Even lower frequencies of methylation have been reported in a heterogeneous HNSCC cohort, where *DAPK1* methylation was detected in 12.5% (4/32) samples (excluding two samples with methylation additionally found in matched normal, indicating background methylation or contamination), and 0% (0/32) of samples demonstrated methylation of *RASSF1A* [36]. In our examination of a single head and neck subsite, using quantitative methodologies, we did not find evidence for frequent methylation of either *DAPK1* or *RASSF1A*. The lower than reported frequency of events detected within our OTSCC cohort was externally validated in the heterogeneous TCGA head and neck cohort (Table 3).

With the use of four orthogonal methodologies, our results demonstrate that one cause for the variability in the frequency of reported methylation events is likely due to the method of assessment of methylation events. Though highly sensitive, techniques such as MSP are well known to be prone to generating false positive results due to the presence of incomplete bisulfite conversion, primer dimer formation, nonspecific primer binding and the subjective nature of determining adequate band intensity representative of a methylated amplicon [11–13]. The limitation of highly sensitive non-quantitative MSP versus quantitative methodology has previously been reported to impact on clinical correlations [98]. Non-quantitative methodology provides no mechanism to account for the detection of low level methylation that can arise from normal infiltrating cells or stromal tissue. In comparison, MS-HRM and SMART-MSP are highly sensitive, semi-quantitative methodologies that provide an advantage for PCR-based assays with the use of melting curve analysis (MCA) [97, 99]. MCA can minimize the inclusion of false positives, allows the quantification of homogeneous methylation, and facilitates the detection of heterogeneous methylation [100].

Gel electrophoresis resolution of generated PCR products from the SMART-MSP analysis permitted the concurrent investigation of whether the use of a non-quantitative methodology may have artificially contributed to the higher frequency of promoter methylation being reported. Using both PCR amplification and MCA to analyze samples for evidence of *DAPK1* promoter methylation, we found that 82% (41/50) of samples demonstrated evidence of methylation. Thus, if reported in isolation, these results would be supportive of the literature. However on quantification, samples demonstrated a median quantity of methylation of 0.0015% (range 0 to 1.320%), with only one sample demonstrating methylation above 1%. Furthermore, gel electrophoresis of products demonstrated similar sized bands for insignificant levels of methylation as well as unamplified DNA, emphasizing the limitations of non-quantitative but highly sensitive methodology. Thus, quantification of methylation levels is an important consideration for the identification of significant methylation events and the interpretation of correlative analyses.

Not surprisingly, quantification of methylation influenced the total number of significant events identified defined by the *a priori* cut-off criteria. However, the use of quantification introduces a number of assumptions. Firstly, the choice of cut-off level is arbitrary and assumes low-level methylation is always irrelevant. The correlation between methylation levels and its impact on gene expression is under investigation, with recent studies demonstrating that the quantity of methylation that alters gene expression differs according to gene and tissue histotype [101]. Our study sought to investigate the significance of methylation as an isolated biomarker, with the use of a cut-off to ensure exclusion of methylation events that arose from normal tissue or were below the sensitivity of the assays used. Secondly, quantitative methodology may not be necessary to identify clinical correlations if the tissue examined demonstrates uniform high levels of methylation that has biological impact.

Our analyses also attempted to account for the potential impact of heterogeneous methylation. Heterogeneous methylation is a complex phenomenon suggestive of epigenetic instability due to incomplete methylation of alleles; however, it is poorly understood and variably detected by existing methodologies [102]. Future research is required to assess methylation levels at the allelic level to enable a comprehensive understanding [103]. Our results demonstrated that if heterogeneous methylation was ignored, then the frequency of reported methylation events for *RUNX3* would have been consistent with the literature. However, pyrosequencing results indicated that a large number of these heterogeneous methylation events were not substantially greater than background levels of methylation. Nevertheless, it is important to note that bisulfite pyrosequencing has limitations quantifying methylation, as methylation is averaged at each CpG site and the amount of templates showing methylation cannot be deduced [14].

External validation of our results was provided by interrogation of the methylation status of the TCGA head and neck samples. While comparison with a heterogeneous HNSCC cohort is difficult, as well as comparisons across different loci, our findings were quite similar in regard to the frequency of methylation events identified overall. The absolute differences in the frequency of methylation events between cohorts may have arisen due to anatomical subsite differences in methylation profiles, the limitation of the smaller size of our cohort overall to detect methylation events, the challenges of identifying and quantifying heterogeneous methylation that may be undetected even with MS-HRM and SMART-MSP, and significantly, the different loci examined within similar regions.

Understanding the specific role of promoter DNA methylation in carcinogenesis is complex and many issues require further research. These include the need to identify which genes according to tissue types that have transcription altered by a specific threshold quantity of methylation, to identify if methylation alters gene expression [101]. The identification of appropriate promoter regions or key CpG dinucleotides that influence transcription for individual genes is also important [104, 105]. Though the use of high throughput methylation analyses have rapidly advanced the field, a thorough understanding of the limitations of each platform is critical. For example, the HM450K probes require hybridization over 50 base pairs, which limits the identification of heterogeneous methylation and informative single CpG sites [104, 106]. Validation and translation of putative methylated biomarkers into clinical practice requires the use of reproducible, appropriately sensitive, quantitative methodology.

Overall, we do not suggest that one method of DNA methylation assessment is necessarily superior. However, quantification of methylation is an important consideration to eliminate very low levels of methylation that are unlikely to confer biological impact, and also to minimize the inclusion of false positives. Ultimately, the choice of methodology is likely to impact the interpretation of the results.

## Conclusions

In conclusion, in a cohort of OTSCC, we demonstrated that there is limited evidence of DNA hypermethylation at significant quantity or frequency for many genes previously reported to be commonly methylated in HNSCC. Our results presented here and recently presented for other cancers [84], suggest that one reason for the overestimation of the frequency of significant methylation events is from the use of non-quantitative methodology. Non-quantitative methylation analyses in the literature for HNSCC should be cautiously interpreted.

## Methods

### Patient information

The patient cohort of 131 OTSCC patients has been described previously [96]. Briefly, patients included consisted of those who we could confirm had OTSCC, had comprehensive clinicopathological and outcome data available, and had pre-treatment FFPE blocks containing invasive squamous cell carcinoma. Of these, 115/131 patient samples had sufficient DNA for bisulfite modification and methylation analyses.

### DNA extraction, bisulfite modification and preparation of control DNA

A hematoxylin and eosin stained slide representative of each pretreatment tumor specimen was labeled by an expert oral pathologist for areas of invasive tumor. Two millimeter representative cores were punched to extract DNA. After deparaffinization with xylene and daily proteinase K digestion over three days at 56°C, DNA was extracted using the QIAamp DNA Blood Mini Kit (Qiagen, Hilden, Germany). A pre-analytical quality-assurance polymerase chain reaction (QA-PCR) [107] was performed that demonstrated the presence of 300 to 600-bp fragments, confirming the quality of DNA was adequate for methylation assessment. For the generation of a dilution series of control DNA standards, fully methylated human genomic DNA (FMD, Merck Millipore, Billerica, US) was purchased, and secondary whole genome amplification (WGA) product was used as unmethylated DNA. The primary WGA product was generated using the Illustra GenomiPhi V2 DNA Amplification Kit (GE Healthcare, Little Chalfont, UK) and then purified with the QIAquick PCR Purification Kit (Qiagen, Hilden, Germany), according to the manufacturers’ instructions. Tumor DNA and control DNA were quantified using the Qubit 2.0 Fluorometer (Life Technologies, Invitrogen, Oregon, US). Bisulfite modification of one microgram of input DNA was performed according to the manufacturer’s instructions with the Methyl Easy *Xceed* kit (Human Genetic Signatures, Sydney, Australia) and eluted into two aliquots of 50 μL, to make a theoretical concentration of 10 ng/μL.

### Methylation sensitive high resolution melting (MS-HRM)

Methylation was assessed in samples initially with MS-HRM [108]. Amplifiable bisulfite modified FMD was normalized against bisulfite modified unmethylated DNA (WGA) using a real-time quantitative PCR assay that amplified a CpG dinucleotide free region of the *COL2A1* gene. Standard dilutions of FMD and WGA were made to 50%, 25%, 10% and 5%. Primers, reaction mixtures and conditions are listed for each promoter region interrogated in Additional file 2: Table S1. MS-HRM analyses were performed on each tumor sample at least in duplicate, with duplicates of the DNA methylation standards, FMD, WGA and non-template control (NTC). Initially, 50 patient samples were assessed for each gene, and then the analysis was extended to the rest of the cohort if any methylation was identified. Heterogeneous methylation was determined as previously described [14, 15, 100]. Briefly, heterogeneous methylation refers to the overall different patterns of methylation that can be observed on any given allele [14], similar to that of genetic mosaicism [109]. When homogeneous methylation is assessed by MS-HRM, melting curves replicate that observed for the FMD or the dilution series of standards. In contrast, when heterogeneous methylation is present, melting curve analysis reveals complex melting patterns as a consequence of heteroduplex formation [14]. If methodology is not specifically chosen to assess the presence of heterogeneous methylation, it will remain undetected. Thus in these instances, the quantity of methylation reported actually represents the mean level of methylation averaged across the entire number of CpG dinucleotides assessed within the amplicon. Importantly, MS-HRM can identify but not quantify heterogeneous methylation.

### Sensitive-melting analysis after real time-methylation specific PCR (SMART-MSP)

We sought to confirm a subset of our findings with a second methodology, and concurrently investigate potential reasons for the discordance between our MS-HRM findings and reported literature. SMART-MSP is a quantitative adaption of MSP, which is readily performed with the added benefit of a melting curve analysis step for quality control [97]. The primers for *DAPK1* were located within the same region interrogated by the MS-HRM primers and produced a 61-bp amplicon. Due to the requirements of primer design, the SMART-MSP primers were located downstream of the MS-HRM primers. PCR cycling and HRM analysis were performed on a Rotor-Gene Q (Qiagen). Each assay was performed with duplicates of the bisulfite modified standard dilution series of FMD and WGA (50%, 25%, 10%, 5%, 1% and 0.1%), tumor samples, NTC and unmodified genomic DNA. The primer sequences, reaction mixtures and conditions are listed in Additional file 2: Table S2.

### Quantification of methylation using Sensitive-melting analysis after real time-methylation specific PCR (SMART-MSP)

The quantity of input DNA of the FMD, WGA and each tumor sample was calibrated against a CpG dinucleotide free region of the *COL2A1* gene. The raw run data for *COL2A1, DAPK1* and *RASSF1A* were generated by the Rotor-Gene Q (Qiagen) and exported into LinRegPCR (version 2012.3.0.0) for the estimation of amplification efficiency, after accounting for bias introduced by variable baseline fluorescence [110]. The proportion of methylated templates was then quantified according to methodology previously described [111].

### Gel electrophoresis analysis

Four microliters of the SMART-MSP product were resolved on a 3.5% agarose gel stained with SYBR Safe (Invitrogen), at 80 mV over 1.5 hours.

### Bisulfite pyrosequencing

The preliminary statistical analysis of the MS-HRM results indicated a significant correlation with worse outcome for samples demonstrating mostly heterogeneous methylation of *RUNX3* and *ABO*. Thus, as MS-HRM cannot quantify heterogeneous methylation, bisulfite pyrosequencing analyses were also performed for these genes. Bisulfite pyrosequencing can quantify the averaged methylation level for each individual CpG dinucleotide within a region of interest [14]. MS-HRM was repeated with the reverse MS-HRM primer containing a 5′-biotin label, and products of duplicates were pooled for bisulfite pyrosequencing [100]. Forward pyrosequencing primers, the interrogated sequence and dispensation order are listed in Additional file 2: Table S3. Analyses were performed on a PyroMark Q96 instrument (Qiagen, Hilden, Germany) with PyroMark Gold Q96 SQA Reagents (Qiagen) according to manufacturer’s instructions, and data was analyzed with the Pyro Q-CpG software (Qiagen, version 1.0.9).

### TCGA data and pre-processing

The TCGA database of head and neck cancers was accessed for publically available methylation data assessed by the Infinium HumanMethylation450 beadchip array (HM450K, Illumina inc., San Diego, US).

Methylation data from Level 1 raw IDAT files were obtained from the heterogeneous TCGA HNSCC samples (http://cancergenome.nih.gov/; HM450K download date 24 June 2013; clinical data download date 22 July 2013). Utilizing R software (version 3.0.1; http://cran.r-project.org/), background correction and probe specific correction with SWAN normalization was performed utilizing Methylumi and Minfi packages found in Bioconductor [112–114]. Probes mapping to common variant SNPs or non-unique mappings were removed from the analysis, leaving 330,557 probes for the downstream analysis [115]. Our MS-HRM amplicons interrogating the *APC, BRCA1, CDH1, CDH13* and *MLH1* genes, overlapped with CpG probes assessed by the HM450K. For the seven genes (*ABO, ATM, DAPK1, RASSF1A, MGMT*, *ERCC1* and *RUNX3*) that did not have overlapping CpGs on the HM450K, the two probes immediately flanking upstream and downstream of our MS-HRM amplicon were assessed. A β-value greater than 0.2 was used to define the presence of significant methylation [116].

### Statistical analysis

In order to account for the sensitivity of assays utilized and background methylation from normal stroma, significant methylation was defined *a priori* as a quantity 10% and greater for MS-HRM, and 15% and greater for pyrosequencing. A higher cut-off was chosen for pyrosequencing to ensure that only significant quantities of methylation were included on the basis that unmethylated DNA controls (WGA) demonstrated quantities of methylation up to 8% at individual CpG sites (see Additional file 2: Table S4).

As heterogeneous methylation can be identified but not quantified on MS-HRM analysis [100], two analyses were performed: one for samples with an estimated level of methylation 10% and greater while excluding samples with heterogeneous methylation, and the second analysis included all heterogeneously methylated samples in the same category.

Following bisulfite pyrosequencing which can quantify the average methylation at individual CpG dinucleotides for both homogenously and heterogeneously methylated samples, correlation with patient outcome was investigated. The analysis included only samples that had 15% and greater mean methylation on pyrosequencing, or for samples with insufficient DNA for pyrosequencing, those with 10% and greater methylation on MS-HRM were also included.

The Kaplan-Meier product-limit method was used to estimate OS and PFS. OS was defined as the time (in years) from the date of diagnosis to the date of death from any cause. PFS was considered only for patients treated with curative intent and was defined as the time (in years) from the date of diagnosis to the date of progression (loco-regional or distant) or death from any cause. A close-out date of 31 January, 2011, was set for the purpose of analysis. Associations between the presence of methylation and survival outcome were assessed using the exact Log-rank test and Cox proportional hazards regression, to estimate hazard ratios (HR) and associated 95% confidence intervals (95% CIs). *P* values <0.05 were regarded as statistically significant.

## Electronic supplementary material

Additional file 1: Figure S1: Representative heat maps for the HM450K probes that overlap MS-HRM amplicons for the TCGA HNSCC cohort. These heat maps represent methylation of A) *APC* and B) *CDH1*, which both demonstrate low levels or no methylation detected for the examined probes. The key in the top left of the figure indicates the β-value, of which the scale of increasing methylation values is indicated from 0% (0) methylation (red) to 100% (0.1) methylated (bright yellow). A β-value >0.2 is considered a significant quantity of methylation. **Figure S2.** Representative heat maps for the HM450K probes that flank MS-HRM amplicons for the TCGA HNSCC cohort. These heat maps represent methylation of A) *DAPK1* and B) *RASSF1A*, which demonstrate low levels or no methylation (red) detected for the examined probes. (DOCX 597 KB)

Additional file 2: Table S1: MS-HRM primers, reaction mixtures, and conditions for selected loci. **Table S2.** SMART-MSP primers, reaction mixtures and conditions for *DAPK1* and *RASSF1A.* **Table S3.** Pyrosequencing primers, interrogated sequence and dispensation order for *ABO* and *RUNX3*. **Table S4.** Methylation levels detected in the unmethylated control DNA (WGA), assessed by pyrosequencing. (DOCX 20 KB)

## Abbreviations

CI: confidence intervals
dHPLC: denaturing high-performance liquid chromatography
ECOG: Eastern Cooperative Oncology Group
FMD: fully methylated DNA
HM450K: Infinium HumanMethylation450 beadchip array
HNSCC: head and neck squamous cell carcinomas
HR: hazards ratio
MCA: melting curve analysis
MS-HRM: methylation sensitive high resolution melting
MS-MLPA: methylation specific multiplex ligation-dependent probe amplification
MSP: methylation-specific PCR
N-category: Node category
NTC: non-template control
OTSCC: oral tongue squamous cell carcinomas
OS: overall survival
PCR: polymerase chain reaction
PFS: progression-free survival
QA-PCR: quality-assurance PCR
RE-MSP: restriction enzyme MSP
SMART-MSP: sensitive-melting analysis after real time-methylation specific PCR
T-category: Tumor category
TCGA: the Cancer Genome Atlas
WGA: whole genome amplified.
