# Supplementary figures and images for: Quantitative methodology is critical for assessing DNA methylation and impacts on correlation with patient outcome
Source: Clin Epigenetics. 2014 Dec 9;6:22. doi: 10.1186/1868-7083-6-22 (PMC4391486; doi:10.1186/1868-7083-6-22)

**Supplementary Figure S1.**


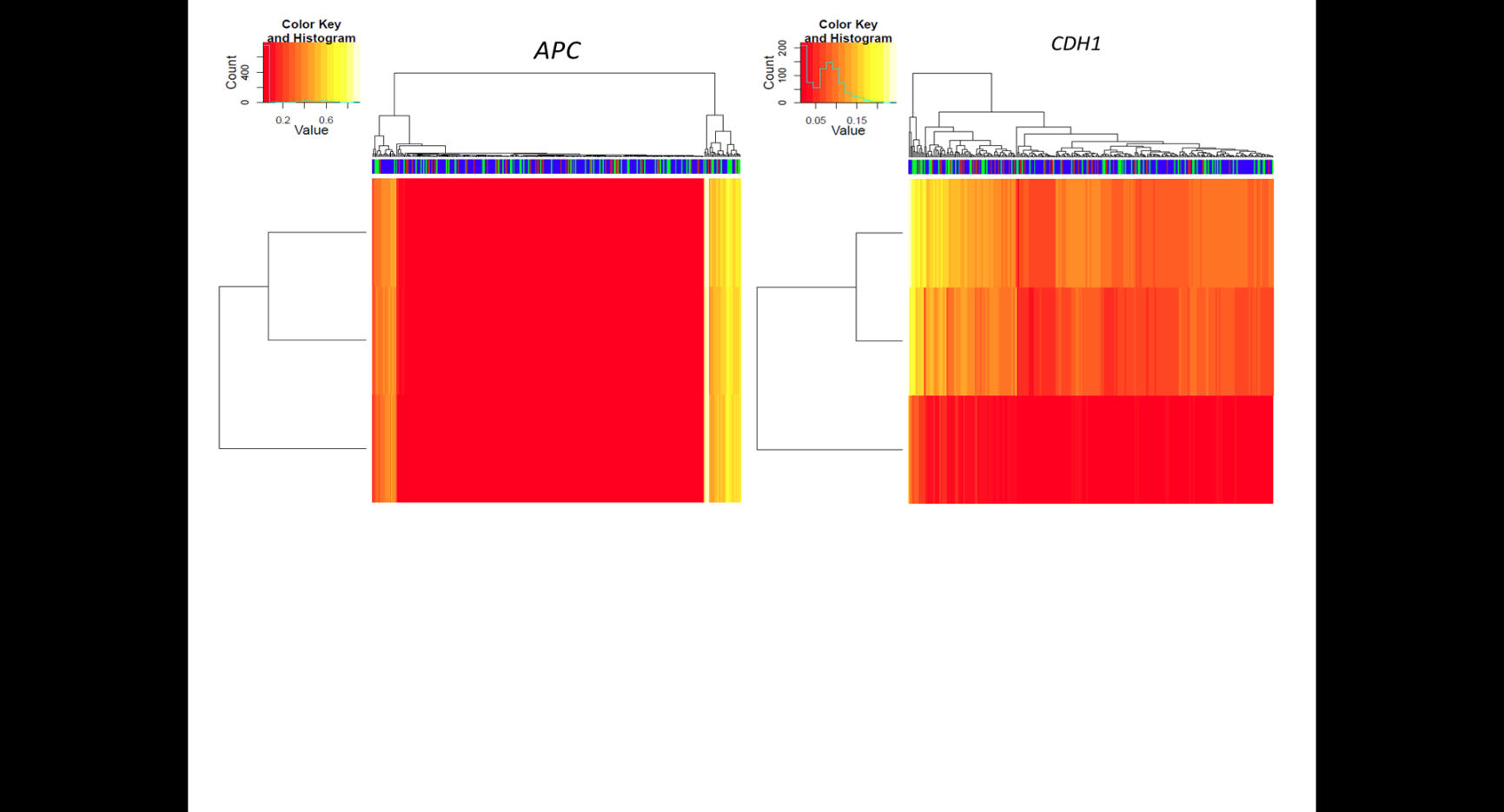


A

B

**Supplementary Figure S2.**


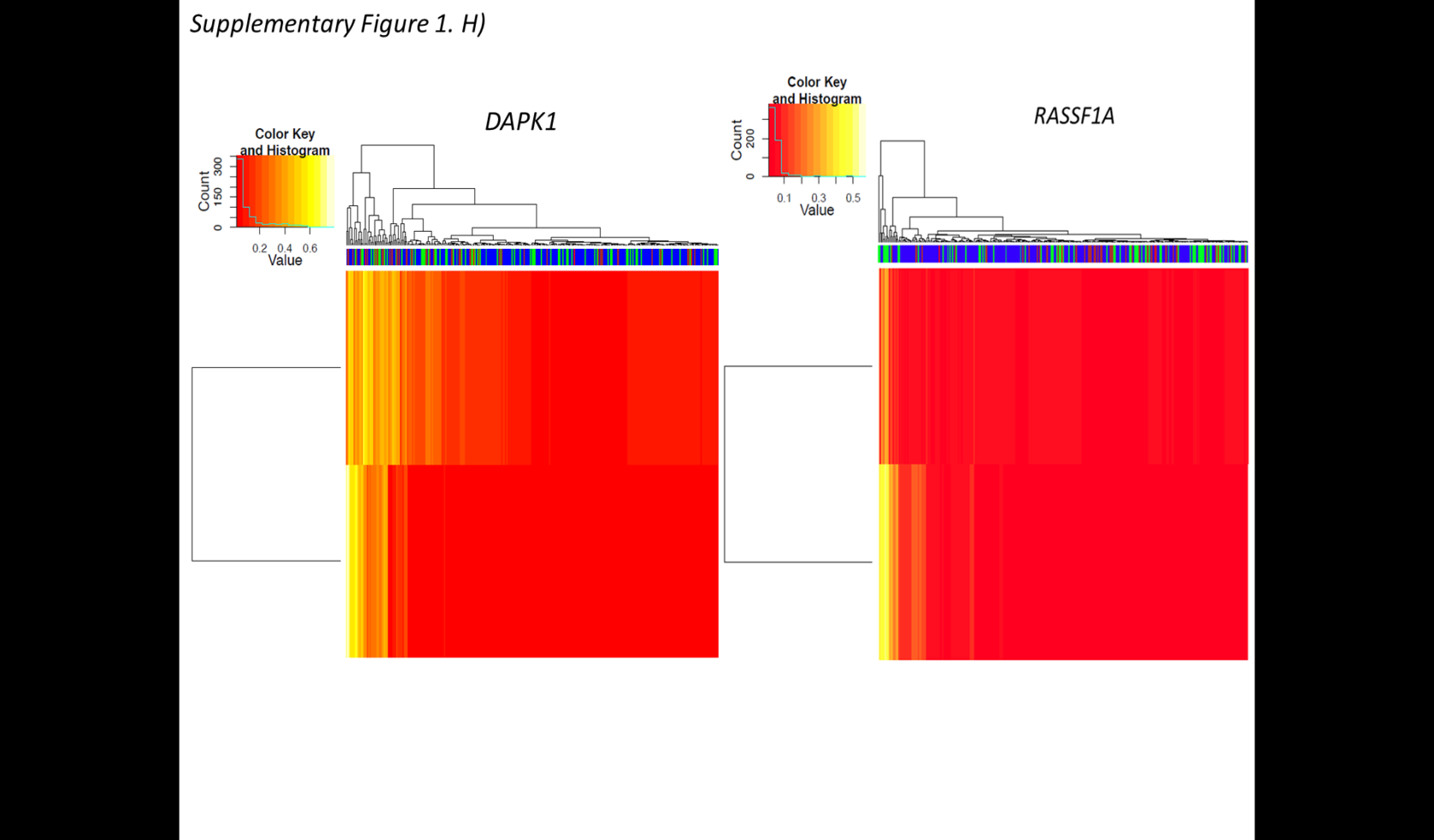


A

B

Supplement: Supplementary file 1 — Additional file 1: Figure S1: Representative heat maps for the HM450K probes that overlap MS-HRM amplicons for the TCGA HNSCC cohort. These heat maps represent methylation of A) APC and B) CDH1, which both demonstrate low levels or no methylation detected for the examined probes. The key in the top left of the figure indicates the β-value, of which the scale of increasing methylation values is indicated from 0% (0) methylation (red) to 100% (0.1) methylated (bright yellow). A β-value >0.2 is considered a significant quantity of methylation. Figure S2. Representative heat maps for the HM450K probes that flank MS-HRM amplicons for the TCGA HNSCC cohort. These heat maps represent methylation of A) DAPK1 and B) RASSF1A, which demonstrate low levels or no methylation (red) detected for the examined probes. (DOCX 597 KB) [file 13148_2014_93_MOESM1_ESM.docx]
