# Supplementary material for: Quantitative methodology is critical for assessing DNA methylation and impacts on correlation with patient outcome
Source: Clin Epigenetics. 2014 Dec 9;6:22. doi: 10.1186/1868-7083-6-22 (PMC4391486; doi:10.1186/1868-7083-6-22)
Supplement: Supplementary file 2 — Additional file 2: Table S1: MS-HRM primers, reaction mixtures, and conditions for selected loci. Table S2. SMART-MSP primers, reaction mixtures and conditions for DAPK1 and RASSF1A. Table S3. Pyrosequencing primers, interrogated sequence and dispensation order for ABO and RUNX3. Table S4. Methylation levels detected in the unmethylated control DNA (WGA), assessed by pyrosequencing. (DOCX 20 KB) [file 13148_2014_93_MOESM2_ESM.docx]

**Supplementary Table S1.**

| **Gene** | **Direction** | **Sequence** | **Reaction Mixture** | **Conditions** |
| --- | --- | --- | --- | --- |
| ***ABO*** | Forward  Reverse | TTCGGGTTTATTTCGTTTTAGGGT  GCAACACCTCGACCATAACTCC | 1 x PCR Buffer, 2.5mM MgCl_2_, 200 µM dNTP, 200nM primer, 5 µM SYTO-9, 0.5U Hotstar Taq. | **Activation**: 95ºC 15mins  **Cycling**: 50x (95ºC for 10secs, 55ºC for 20secs, 72ºC for 20secs)  **Inactivation**: 97ºC, 1min  **HRM**: 68-97ºC (0.2ºC/step) |
| ***APC*** | Forward  Reverse | CGGGGTTTTGTGTTTTATTG  TCCAACGAATTACACAACTAC | 1 x PCR Buffer, 4mM MgCl_2_, 200 µM dNTP, 200nM forward primer, 300nM reverse primer,  5µM SYTO-9, 0.5U Hotstar Taq. | **Activation**: 95ºC 15mins  **Cycling**: 55x (95ºC for 10secs, 55ºC for 20secs, 72ºC for 25secs)  **Inactivation**: 97ºC, 1min  **HRM**: 70-90ºC (0.2ºC/step) |
| ***ATM*** | Forward  Reverse | GTTTGCGTTAWGTTTATTAATGGTT*  ACTTCCGTCCTCAAACTTAAAA | 1 x PCR Buffer, 3mM MgCl_2_, 200 µM dNTP, 200nM primer, 5µM SYTO-9, 0.5U Hotstar Taq. | **Activation**: 95ºC 15mins  **Cycling**: 45x (95ºC for 20secs, 52ºC for 30secs, 72ºC for 20secs)  **Inactivation**: 97ºC, 1min  **HRM**: 65-95ºC (0.2ºC/step) |
| ***BRCA1*** | Forward  Reverse | TTGTTGTTTAGCGGTAGTTTTTTGGTT  CAATCGCAATTTTAATTTATCTATAATTCC | 1 x PCR Buffer, 4mM MgCl_2_, 200 µM dNTP, 250nM primer, 5µM SYTO-9, 0.5U Hotstar Taq. | **Activation**: 95ºC 15mins  **Cycling**: 55x (95ºC for 10secs, 58ºC for 10secs, 72ºC for 20secs)  **Inactivation**: 97ºC, 1min  **HRM**: 70-85ºC (0.2ºC/step) |
| ***CDH1***  ***(CDH1)*** | Forward  Reverse | GAGTTTGCGGAAGTTAGTTTAGATTTTAG  CGACTCCAAAAACCCATAACTAACC | 1 x PCR Buffer, 3mM MgCl_2_, 200 µM dNTP, 200nM primer, 5µM SYTO-9, 0.5U Hotstar Taq. | **Activation**: 95ºC 15mins  **Cycling**: 50x (95ºC for 15secs, 58ºC for 15secs, 72ºC for 20secs)  **Inactivation**: 97ºC, 1min  **HRM**: 65-95ºC (0.2ºC/step) |
| ***CDH13*** | Forward  Reverse | TTGGTTTTTACGGAAAATATGTTTAGTGTA  AATTCTCGACTACATTTTATCCGACTAAAA | 1 x PCR Buffer, 3mM MgCl_2_, 200 µM dNTP, 300nM forward primer, 400nM reverse primer,  5µM SYTO-9, 0.5U Hotstar Taq. | **Activation**: 95ºC 15mins  **Cycling**: 55x (95ºC for 10secs, 55ºC for 20secs, 72ºC for 25secs)  **Inactivation**: 97ºC, 1min  **HRM**: 65-90ºC (0.2ºC/step) |
| ***DAPK1*** | Forward  Reverse | GTTAGTTCGTTTGTAGGGTTTTTATTGGT  GCCGACCCCAAACCCTACC | 1 x PCR Buffer, 2.5mM MgCl_2_, 200 µM dNTP, 200nM primer, 5µM SYTO-9, 0.5U Hotstar Taq. | **Activation**: 95ºC 15mins  **Cycling**: 55x (95ºC for 10secs, 56ºC for 20secs, 72ºC for 20secs)  **Inactivation**: 97ºC, 1min  **HRM**: 65-99ºC (0.2ºC/step) |
| ***ERCC1*** | Forward  Reverse | TGTAAAACGACGGCCAGTGAGTCGTTTTTTTTTATTWGGGTTTTTTTG*  CAGGAAACAGCTATGACCCGCCCGCCTCTAAACTTAACC | 1 x PCR Buffer, 2.5mM MgCl_2_, 200 µM dNTP, 250nM primer, 5µM SYTO-9, 0.5U Hotstar Taq. | **Activation**: 95ºC 15mins  **Cycling**: 50x (95ºC for 10secs, 63ºC for 20secs, 72ºC for 20secs)  **Inactivation**: 97ºC, 1min  **HRM**: 65-95ºC (0.2ºC/step) |
| ***MGMT*** | Forward  Reverse | GCGTTTCGGATATGTTGGGATAGT  AACGACCCAAACACTCACCAAA | 1 x PCR Buffer, 4mM MgCl_2_, 200 µM dNTP, 250nM primer, 5 µM SYTO-9, 0.5U Hotstar Taq. | **Activation**: 95ºC 15mins  **Cycling**: 45x (95ºC for 10secs, 55ºC for 20secs, 72ºC for 20secs)  **Inactivation**: 97ºC, 1min  **HRM**: 65-95ºC (0.2ºC/step) |
| ***MLH1***  ***(MLH1)*** | Forward  Reverse | AGTTTTTAAAAACGAATTAATAGGAAGAG  ACTACCCGCTACCTAAAAAAATATAC | 1 x PCR Buffer, 3.5mM MgCl_2_, 200 µM dNTP, 200nM forward primer, 300nM reverse primer, 5 µM SYTO-9,  0.5U Hotstar Taq. | **Activation**: 95ºC 15mins  **Cycling**: 55x (95ºC for 10secs, 55ºC for 20secs, 72ºC for 20secs)  **Inactivation**: 97ºC, 1min  **HRM**: 65-95ºC (0.2ºC/step) |
| ***RASSF1A*** | Forward  Reverse | TCGGGTTTTATAGTTTTTGTATTTAGGTTTT  CCTCCCCCAAAATCCAAACTAA | 1 x PCR Buffer, 3mM MgCl_2_, 200 µM dNTP, 300nM forward primer, 200nM reverse primer, 5 µM SYTO-9, 0.5U Hotstar Taq. | **Activation**: 95ºC 15mins  **Cycling**: 55x (95ºC for 10secs, 55ºC for 20secs, 72ºC for 30secs)  **Inactivation**: 97ºC, 1min  **HRM**: 65-95ºC (0.2ºC/step) |
| ***RUNX3*** | Forward  Reverse | GTTTCGGGTTTCGTATTTATTTTGAAGG  GACAACCCCAACTTCCTCTACTC | 1 x PCR Buffer, 3mM MgCl_2_, 200 µM dNTP, 200nM primer, 5 µM SYTO-9, 0.5U Hotstar Taq. | **Activation**: 95ºC 15mins  **Cycling**: 50x (95ºC for 15secs, 55ºC for 15secs, 72ºC for 20secs)  **Inactivation**: 97ºC, 1min  **HRM**: 65-95ºC (0.2ºC/step) |

**Supplementary Table S2.**

| **Gene** | **Direction** | **Sequence** | **Reaction Mixture** | **Conditions** |
| --- | --- | --- | --- | --- |
| ***DAPK1*** | Forward  Reverse | AGGATAGTCGGATCGAGTTAACGTC  TTACCGAATCCCCTCCGCGA | 1 x PCR Buffer, 2.5mM MgCl_2_, 200 µM dNTP, 200nM primer, 5 µM SYTO-9, 0.5U Hotstar Taq. | **Activation**: 95ºC 15mins  **Cycling**: 55x (95ºC for 5secs, 60ºC for 10secs, 72ºC for 10secs)  **Inactivation**: 97ºC, 1min  **HRM**: 72-85ºC (0.2ºC/step) |
| ***RASSF1A*** | Forward  Reverse | CGTTCGGTTCGCGTTTGTTAGC  CATAACCCGATTAAACCCGTACTTCG | 1 x PCR Buffer, 3mM MgCl_2_, 200 µM dNTP, 300nM forward primer, 200nM reverse primer, 5µM SYTO-9, 0.5U Hotstar Taq. | **Activation**: 95ºC 15mins  **Cycling**: 55x (95ºC for 10secs, 65ºC for 20secs, 72ºC for 20secs)  **Inactivation**: 97ºC, 1min  **HRM**: 65-95ºC (0.2ºC/step) |

**Supplementary Table S3.**

| **Gene** | **Sequence** | **Interrogated sequence** | **Dispensation order** |
| --- | --- | --- | --- |
| ***ABO***  **Forward** | TTTATTTCGTTTTAGGGT | TTCGGGTTTATTTCGTTTTAGGGTCGTCGGGCGGAAGGCGGAGGTCGAGATTAGACGCGGAGTTATGGTCGAGGTGTTGC | GTCTGTCAGTCGATGTCGATGTCGAGATAGTATCAGTCG |
| ***RUNX3***  **Forward** | CGTATTTATTTTGAAGG | GTTTCGGGTTTCGTATTTATTTTGAAGGCGACGGGTAGCGTTTTGTTGTAGCGTTAGTGCGAGGGTAGTACGGAGTAGAGGAAGTTGGGGTTGTC | ATCGTATCGTATGTCGTTGTGTATGTCGTAGTAGTCGAGTAGTGATCGA |

**Supplementary Table S4.**

| **WGA** | **CpG 1** | **CpG 2** | **CpG 3** | **CpG 4** | **CpG 5** | **CpG 6** | **CpG 7** |
| --- | --- | --- | --- | --- | --- | --- | --- |
| ***ABO* control 1** | 1 | 3 | 2 | 4 | 8 | 8 | 4 |
| ***ABO* control 2** | 2 | 3 | 2 | 2 | 4 | 4 | 2 |
| ***ABO* control 3** | 2 | 2 | 2 | 2 | 4 | 4 | 0 |
| ***ABO* control 4** | 2 | 2 | 1 | 0 | 1 | 2 | 0 |
| ***ABO* control 5** | 0 | 5 | 2 | 3 | 5 | 5 | 0 |
| ***ABO* control 6** | 3 | 3 | 2 | 4 | 5 | 5 | 0 |
| ***RUNX3* control 1** | 5 | 0 | 2 | 4 | 5 | 7 | NA |
| ***RUNX3* control 2** | 5 | 0 | 0 | 7 | 0 | 5 | NA |
| ***RUNX3* control 3** | 6 | 0 | 0 | 0 | 0 | 0 | NA |
| ***RUNX3* control 4** | 4 | 0 | 0 | 3 | 0 | 4 | NA |
